# Mutations of *CNTNAP1* led to defects in neuronal development

**DOI:** 10.1172/jci.insight.135697

**Published:** 2020-11-05

**Authors:** Wanxing Li, Lin Yang, Chuanqing Tang, Kaiyi Liu, Yulan Lu, Huijun Wang, Kai Yan, Zilong Qiu, Wenhao Zhou

**Affiliations:** 1 Division of Neonatology,; 2 Key Laboratory of Birth Defects,; 3 Division of Endocrinology, Genetics and Metabolic Disease, and; 4 Stem Cell Research Center, Institute of Pediatrics, Children’s Hospital, Fudan University, Shanghai, China.; 5 Institute of Neuroscience, State Key Laboratory of Neuroscience,; 6 CAS Center for Excellence in Brain Science and Intelligence Technology,; 7 Shanghai Center for Brain Science and Brain-Inspired Intelligence Technology,; 8 Chinese Academy of Sciences, and; 9 National Clinical Research Center for Aging and Medicine, Huashan Hospital, Fudan University, Shanghai, China.; 10 Key Laboratory of Neonatal Diseases, Ministry of Health, Children’s Hospital of Fudan University, Shanghai, China.

**Keywords:** Genetics, Neuroscience, Neurodevelopment

## Abstract

Mutations of *CNTNAP1* were associated with myelination disorders, suggesting the role of *CNTNAP1* in myelination processes. Whether *CNTNAP1* may have a role in early cortical neuronal development is largely unknown. In this study, we identified 4 compound heterozygous mutations of *CNTNAP1* in 2 Chinese families. Using mouse models, we found that *CNTNAP1* is highly expressed in neurons and is located predominantly in MAP2^+^ neurons during the early developmental stage. Importantly, *Cntnap1* deficiency results in aberrant dendritic growth and spine development in vitro and in vivo, and it delayed migration of cortical neurons during early development. Finally, we found that the number of parvalbumin^+^ neurons in the cortex and hippocampus of *Cntnap1*^–/–^ mice is strikingly increased by P15, suggesting that excitation/inhibition balance is impaired. Together, this evidence elucidates a critical function of *CNTNAP1* in cortical development, providing insights underlying molecular and circuit mechanisms of *CNTNAP1*-related disease.

## Abstract 2

Deficiency of CNTNAP1 causes severe cortical developmental deficits, leading to human lethal perinatal symptoms.

## Introduction

The *CNTNAP1* gene encodes contactin-associated protein (CNTNAP1), which is a cell-adhesion protein belonging to the neurexin superfamily (1). This family also includes CNTNAP2, CNTNAP3, CNTNAP4, and CNTNAP5. Several animal studies have found that CNTNAP1 and CNTNAP2 are essential for the axoglial interactions required for normal saltatory conduction in the CNS and peripheral nervous system (PNS) (2–5). Recently, the CNTNAP family has been found to play roles in brain development. CNTNAP2–5 are associated with autism spectrum disorders (ASDs) (6–9). CNTNAP2 is also critical for neurite and synapse development, cortical neuronal migration, and the development of GABAergic interneurons (7, 8). CNTNAP3 deficiency results in an imbalance of excitatory and inhibitory synapse development, causing ASD-like behaviors in mice (9). CNTNAP4 is expressed in NPCs and inhibits neurogenesis (10). Furthermore, CNTNAP4 plays a vital role in regulating the neurotransmission of GABAergic neurons (11).

CNTNAP1 is highly expressed in radial glial cells (RGCs) in the early neurogenic period and plays a decisive role in the timing of neuron and astrocyte generation (12). CNTNAP1 has also been found to bind Cntn1 (13, 14), neurofascin (2, 15, 16), and native prion protein (17), which are involved in neurite outgrowth (18), neuronal migration (19), dendritic branching (20, 21), and synaptogenesis (22), thereby allowing inferences about its potential functions in early brain development.

In the current study, we reported 2 patients diagnosed as neuropathy caused by potentially novel *CNTNAP1* mutations. To date, only 29 *CNTNAP1*-related neuropathy cases were reported on PubMed since 2014. All of them were diagnosed as congenital hypomyelinating neuropathy (OMIM #618186) with/without lethal congenital contracture syndrome (OMIM #616286). The broad phenotypic spectrum that resulted from the *CNTNAP1* mutations included prenatal onset (fatal akinesia and polyhydramnios) and lethal neonatal symptoms (severe respiratory distress leading to tracheotomy,ventilator dependency, and hypotonia). Most of the patients who survived to infancy (one-fourth died within 1 month) were found to have seizures and brain atrophy (23–32). However, the common clinical phenotypes, such as prenatal onset with fetal akinesia, severe respiratory distress immediately after birth, and very early lethality, are inconsistent with typical hypomyelinating leukodystrophy. It is well known that one of the major clinical features of typical hypomyelinating leukodystrophies, such as Alexander disease (OMIM # 203450), Canavan disease (OMIM #271900), and Pelizaeus-Merzbacher disease (PMD) (OMIM # 312080), is a developmental regression after an uneventful pregnancy and a period of normal growth and development. This is in line with the most rapid and dramatic period of human CNS myelination within the first 2 years of postnatal life (33, 34). Notably, other CNTNAP family–related (CNTNAP2–5–related) disorders predominantly feature the neuronal manifestations mentioned above. The combined observations suggest that our existing understanding of the pathology of *CNTNAP1*-related disorders likely underestimates the function of *CNTNAP1* in early neuronal development, which begins before myelination.

The de novo mutation Asp1140Tyr (D1140Y) of our patient significantly decreased the protein levels of *CNTNAP1* in vitro. It suggested that the developmental defects might be attributable to *CNTNAP1* deficiency. Therefore, we knocked down *Cntnap1* expression in primary neurons in vitro and in the excitatory neurons of developing cortex to examine whether *Cntnap1* may play roles in early neuronal development. We found that *Cntnap1* knockdown (KD) caused a delay in neuronal migration and that KD neurons also exhibited aberrantly branching processes. We further confirmed these neuronal defects in *Cntnap1*-KO mice. We also found that *Cntnap1* deficiency results in an increase of dendritic spine density in vivo. Strikingly, an altered number of parvalbumin^+^ (PV^+^) and vasoactive intestinal peptide^+^ (VIP^+^) interneurons was revealed in *Cntnap1*-KO mice brain. These results suggest that *CNTNAP1* has a potentially novel function in neuronal development at an early age, explaining neuronal pathology of the *CNTNAP1*-related phenotypes in human.

## Results

### Clinical report.

**Figure 1:**
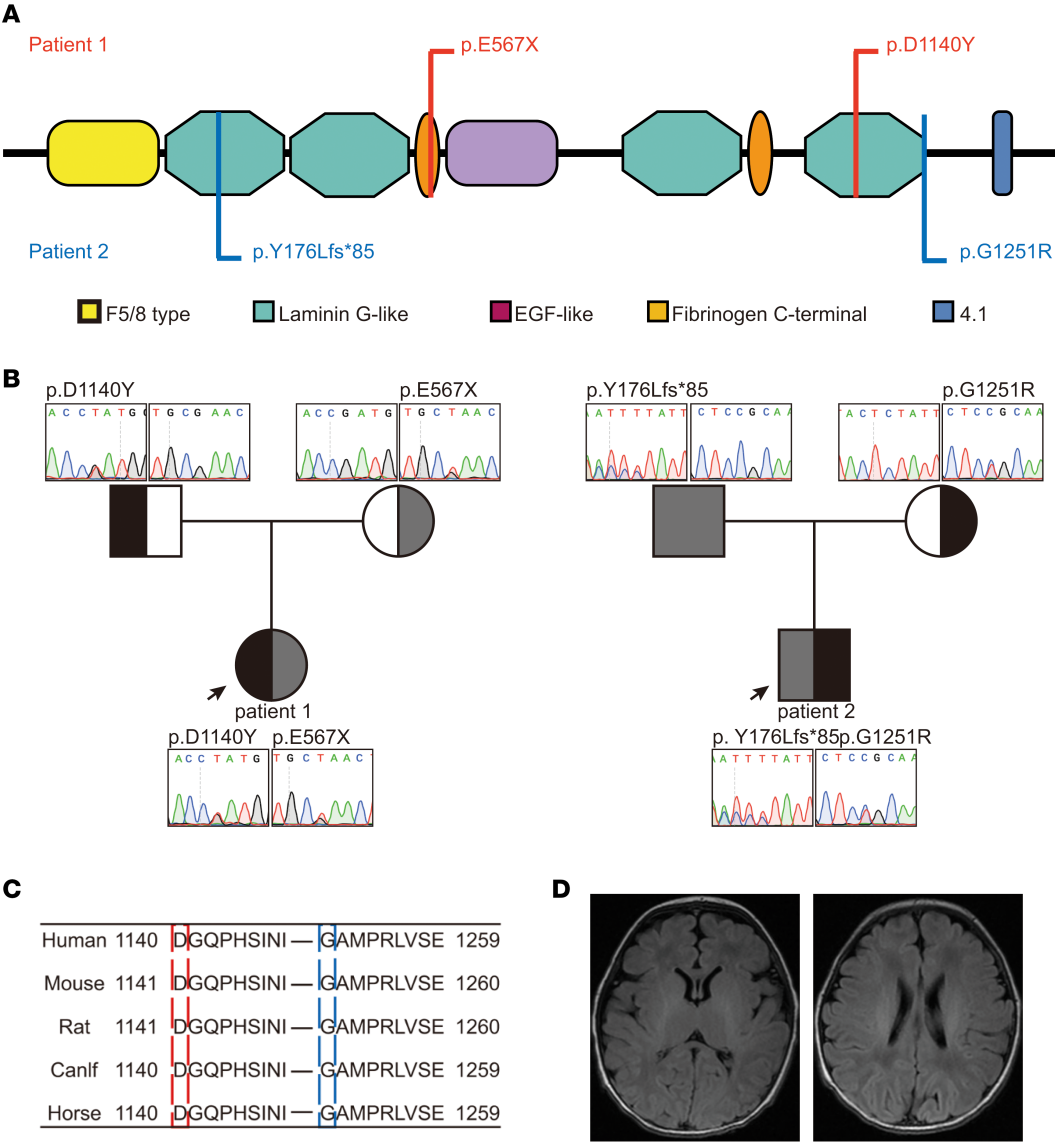
Identification of variants in the *CNTNAP1* gene in 2 unrelated families. (**A**) Schematic representation of the *CNTNAP1* domain and the approximate location of the potentially novel variants. Patient 1 exhibited c.G1699T.p.E567* and c.G3418T.p.D1140Y (in red). Patient 2 exhibited c.526insT.p.Y176Lfs*85 and c.G3751A.p.G1251R (in blue). (**B**) Sanger sequencing confirmed that patient 1 paternally inherited p.D1140Y and maternally inherited p.E567*, and that patient 2 paternally inherited p.Y176Lfs*85 and maternally inherited p.G1251R. (**C**) The orthologous alignment of the p.D1140Y and p.G1251R. The aspartic acid at position 1140 and glycine at position 1251 are highly conserved across species (https://www.uniprot.org/). (**D**) Brain MR (FLAIR) of patient 1 at 10 days old showed a wide temporal extracerebral space.

Both patients were the first child (G1P1) of healthy and nonconsanguineous parents from 2 unrelated Chinese families. Patient 1 was a female and born in the 40th gestational week by caesarean section after a pregnancy with polyhydramnios (fluid volume was 1800 mL) and decreased fetal movement. She was born with normal birth weight of 3550 g and normal occipitofrontal circumference (OFC). The Apgar score was 8-8-8. She was recruited to our hospital due to severe respiratory distress. Generalized hypotonia immediately occurred after birth, resulting in mechanical ventilation support. Neurological examination revealed hypertonicity with less voluntary activity. Chest x-ray showed neonatal respiratory distress syndrome (NRDS). Sensory and motor nerve–involved peripheral neuropathy (PNP) and hypomyelination axonopathy were observed by electromyogram (EMG). MRI of the brain at 10 days old suggested a wide temporal extracerebral space with a normal electroencephalogram (EEG) (Figure 1D). The karyotype was normal. She was diagnosed as PNP, laryngeal dysplasia, neonatal pneumonia, neonatal encephalopathy, and congenital hypotonia. An artificial nasal was used to assist breathing since day 7 of life. Because of her feeding difficulty, nasal feeding was initiated on day 7 of life. She was discharged at the age of 7 weeks. Follow-ups at 8 months showed no acquisition of developmental milestones and an arrest of head growth (OFC was 41 cm, *Z* score = –2.27). Her family history was unremarkable.

Patient 2 was born at full term by caesarean section after a pregnancy with polyhydramnios. He was born with a birth weight of 4400 g. He was recruited to our hospital due to neonatal asphyxia, neonatal pneumonia, and neonatal hypotonia. Follow-ups showed developmental delay, hypertonia, and muscle weakness at 1 year old and recurrent seizures at 2 years old with a poor response to sodium valproate (VPA) treatment, as well as video-electroencephalography (VEEG) presenting multiple sharp waves, spike waves, sharp slow waves, and poly-spike waves during waking and sleeping. His parents are healthy, but his maternal uncle had a history of epilepsy.

### Identification of potentially novel variants in the CNTNAP1 gene in 2 unrelated families.

Whole-exome sequencing (WES) was performed in the 2 trio-families. We found that an average of 99.85% of the target region was covered (an average of 99.28% was covered at 10×, and an average of 97.78% was covered at 20×). Twelve variants of 9 genes (*CNTNAP1*, *DNAH6*, *EVC2*, *FLG*, *FOXRED1*, *MAP3k15*, *RS1*, *TBX2*, and *TTN*) were found in trio-1, and 6 variants of 4 genes (*CNTNAP1*, *COLQ*, *NBPF1*, *PIEZO1*) were found in trio-2 after filtering variants with low quality or those that appeared in public (ExAC or 1000 genome) or our inner control databases. No copy number variants (CNVs) of phenotype-related genes were identified. We screened these candidate genes for their relevance to the phenotypes of our 2 patients, and *CNTNAP1* was considered to be the most significant phenotype-related candidate gene for both probands.

Both patients were compound heterozygous for the *CNTNAP1* gene (Figure 1, A and B). In patient 1, we identified a maternal stop-gain mutation (c.1699G>T [Glu567*]) in exon 11 and a paternal missense mutation (c.3418G>T [Asp1140Tyr]) in exon 20. In patient 2, we identified a paternal truncating mutation (c.526insT [Tyr176Leufs*85]) in exon 5 and a maternal missense mutation (c.3751G>A [Gly1251Arg]) in exon 22. All 4 variants in the probands and their parents were confirmed by Sanger sequencing (Figure 1B). The orthologous alignment of the 4 substitutions (Tyr in position 176, Glu in 567, Asp in 1140, and Gly in 1251) is highly conserved across species (https://www.uniprot.org/) (Figure 1C). They have not been reported in the ExAC, 1000 genome databases, and our inner databases. Therefore, we considered these variants to be rare mutations. In addition to Glu567* and Tyr176Leufs*85 causing protein truncation, both point mutations (c.3418G>T [Asp1140Tyr] and c.3751G>A [Gly1251Arg]) were predicted to be deleterious according to Polyphen-2 and MutationTaster (c.3418G>T scored 0.993 and 1, while c.3751G>A scored 0.986 and 1, respectively).

**Figure 2:**
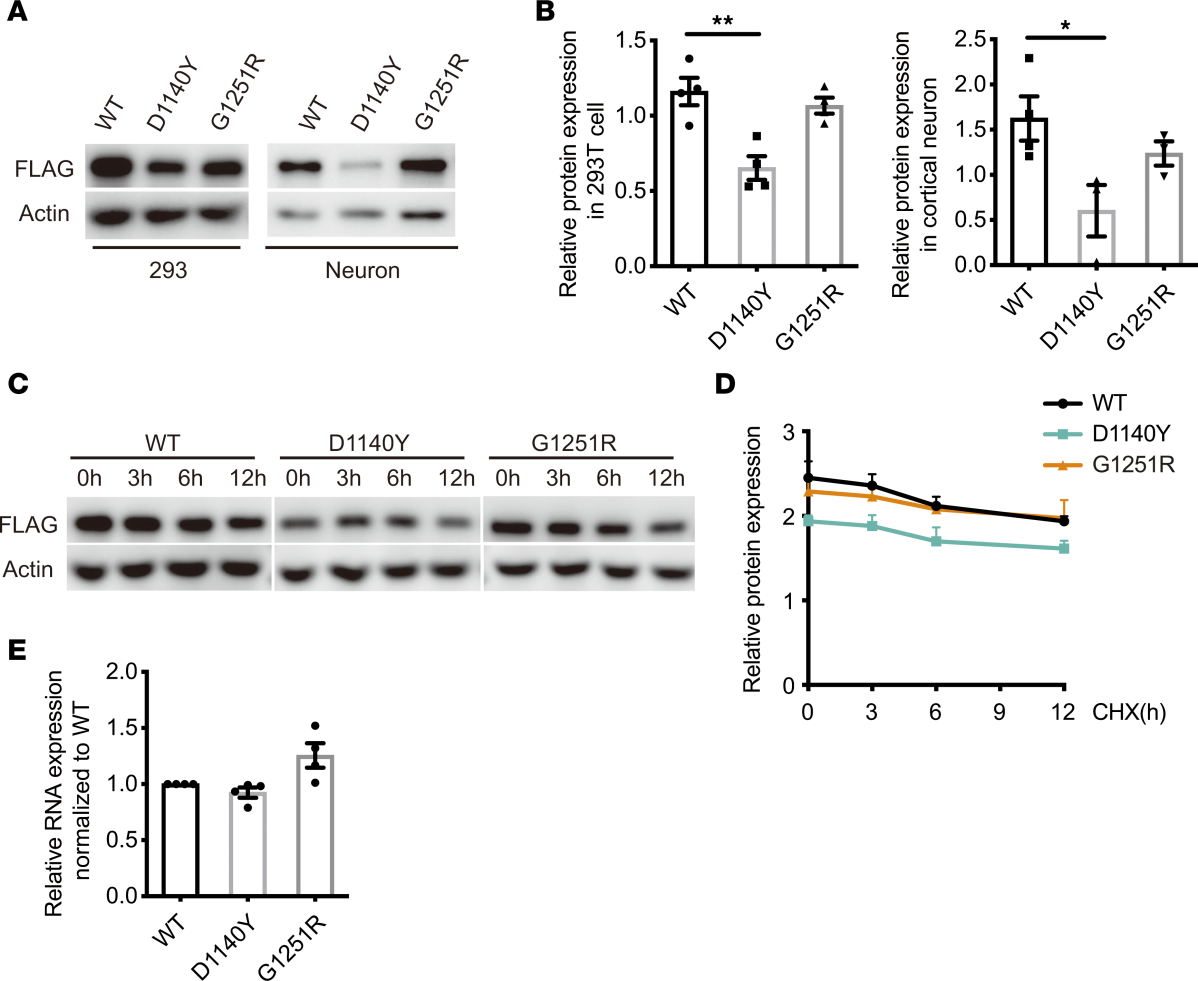
D1140Y and G1251R expression levels in 293T cells and mouse primary cortical neurons. (**A**) Representative Western blots of 293T cells and mouse primary cortical neurons transfected with Flag-tagged CNTNAP1-WT, CNTNAP1-D1140Y, and CNTNAP1-G1251R. β-Actin was used as a loading control. (**B**) Relative protein expression of Flag-tagged CNTNAP1-WT, CNTNAP1-D1140Y, and CNTNAP1-G1251R in 293T cells and mouse primary cortical neurons (represents the ratio of Flag in the upper panel to actin in the lower panel). The band intensities were determined by ImageJ. Statistical significance was evaluated by ordinary 1-way ANOVA: **P = 0.0028; *P = 0.0413. Data are shown as mean ± SEM. All data were collected from 4 independent experiments. (**C**) Representative Western blots of 293T cells transfected with Flag-tagged CNTNAP1-WT, CNTNAP1-D1140Y, and CNTNAP1-G1251R and treated with cycloheximide (CHX; 20 μg/mL) for 0, 3, 6, and 12 hours. (**D**) Relative protein expression of Flag-tagged CNTNAP1-WT, CNTNAP1-D1140Y, and CNTNAP1-G1251R in 293T cells (represents the ratio of Flag in the upper panel to actin in the lower panel). The band intensities were determined by ImageJ. Statistical significance was evaluated by ordinary 1-way ANOVA. Data are shown as mean ± SEM. All data were collected from 3 independent experiments. (**E**) Relative RNA expression of Flag-tagged CNTNAP1-WT, CNTNAP1-D1140Y, and CNTNAP1-G1251R in mouse primary cortical neurons. The level was normalized to that of WT group. Statistical significance was evaluated by ordinary 1-way ANOVA. Data are shown as mean ± SEM. All data were collected from 3 independent experiments.

To study the protein properties of the D1140Y and G1251R mutants, we constructed overexpression vectors expressing human CNTNAP1-WT (WT), CNTNAP1-D1140Y, and CNTNAP1-G1251R cDNAs. Plasmids were transfected into 293T cells, and primary cortical neurons were isolated from embryonic C57BL/6J mice. We found that the protein level of the D1140Y mutant was significantly reduced compared with that of WT protein in 293T cells and primary cortical neurons (Figure 2, A and B). However, there was no significant difference between the levels of WT and the G1251R mutant, which was consistent with the phenotype severity of our 2 patients.

However, the decrease in the protein level was not caused by perturbed protein stability, since we measured the protein level after blocking protein synthesis by cycloheximide (CHX) treatment, suggesting that the protein stability of WT CNTNAP1 and the D1140Y and G1251R mutants was not significantly different in cultured neurons in vitro (Figure 2, C and D). We further collected the total RNA of neurons expressing human WT, CNTNAP1-D1140Y, and CNTNAP1-G1251R. Through real-time PCR, no significant difference of RNA expression level was found between 3 groups (Figure 2E).

### Clinical and genetic findings in our patients and previously reported patients with CNTNAP1 variants.

According to the summary of findings in all 31 cases with *CNTNAP1* variants, including ours and those published on PubMed, we found that 100% had hypotonia and hypomyelination, which can be explained by the well-studied function of CNTNAP1 in axoglial myelination (Table I). However, we also found that 94.1% had brain atrophy. Considering that MRI performed within 1 month showed that 3 patients had brain atrophy, we believed that the brain atrophy was not completely secondary to hypomyelination. A total of 93.5% of newborns needed mechanical ventilation support after birth due to respiratory distress. A total of 84.6% presented fatal akinesia, and 74.2% presented polyhydramnios that may have been caused by fetal akinesia and malfunction of the nervous system; 54.5% had seizures. In addition, 25.8% died within 1 month after birth, and 81.3% required tracheotomy and mechanical ventilation (Table 1). The need for mechanical ventilation indicated that survival without aggressive medical intervention was very difficult for most patients.

It has always been considered that mutations of *CNTNAP1* gene might cause CNS hypomyelination, and its clinical manifestations are developmental regression and loss of obtained motor abilities. The onset of this kind of diseases begins in infants or older because the developmental period of myelination begins around 2 years of age. However, none of those mentioned phenotypes above matched the typical characteristics of CNS hypomyelination. Therefore, a potentially novel role for *CNTNAP1* in neuronal development during the perinatal period might be suggested.

### Expression of Cntnap1 during cerebral cortex maturation.

**Figure 3:**
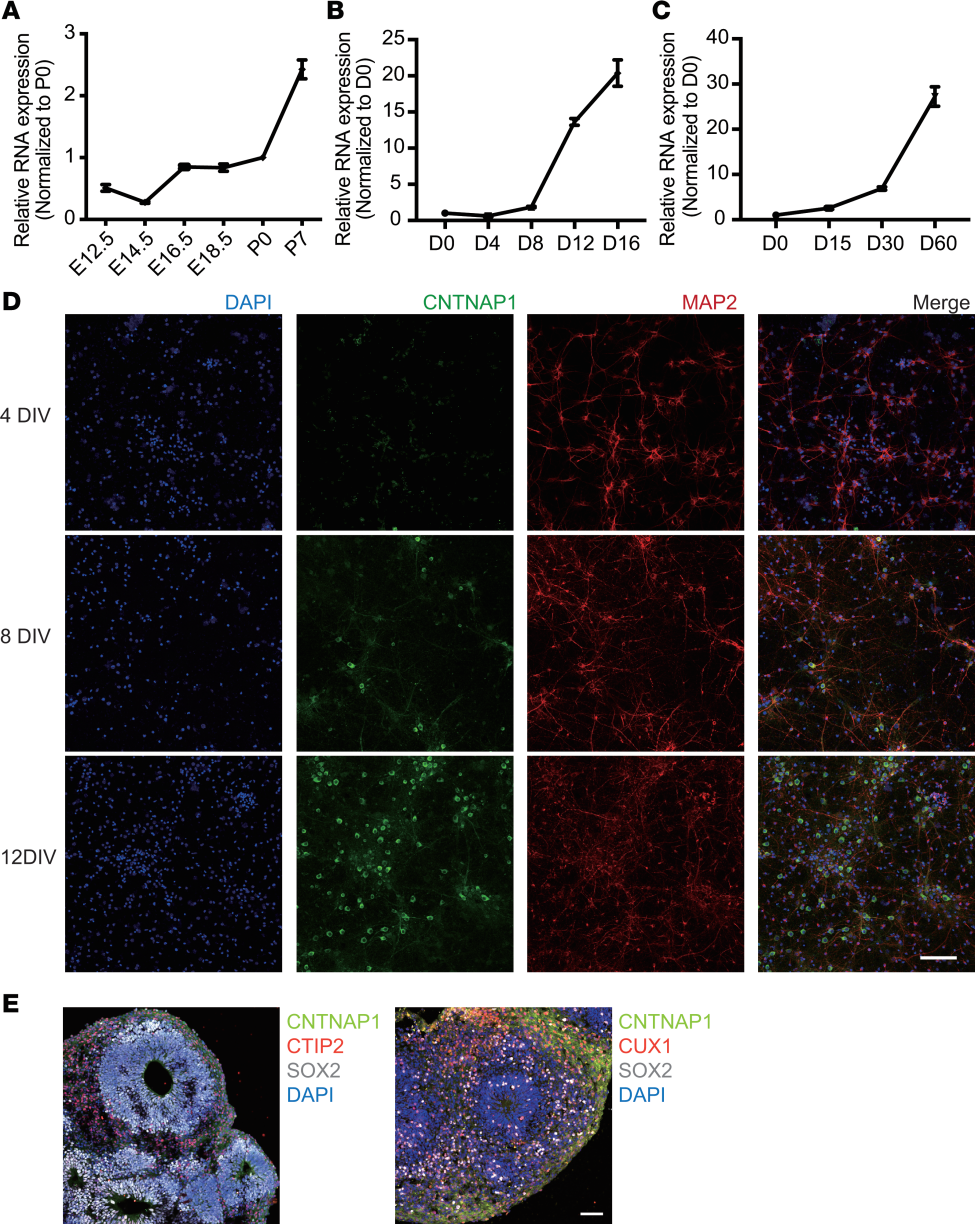
Expression of *Cntnap1* during cerebral cortex development. (**A**) *Cntnap1* mRNA expression in the developing mouse cerebral cortex (*n* = 4 at each time point), as determined by real-time PCR. The level was normalized to that of P0 mice. Data are shown as mean ± SEM. (**B**) *Cntnap1* mRNA expression in the mouse primary cortical neurons cultured in vivo (*n* = 3 at each time point), as determined by real-time PCR. The level was normalized to that at 0DIV. Data are shown as mean ± SEM. (**C**) *Cntnap1* mRNA expression in human iPSC–derived forebrain organoids (*n* = 3 at each time point), as determined by real-time PCR. The level was normalized to that at 0DIV. Data are shown as mean ± SEM. (**D**) Representative images of mouse primary cortical neurons in vitro with double immunochemistry for CNTNAP1 (green) and MAP2 (red). Scale bar: 100 μm. (**E**) Representative images of human iPSC-–derived forebrain organoid by day 30 (left) and day 60 (right). Organoid by day 30 was immunostained with Cntnap1 (green), CTIP2 (red), and SOX2 (gray). Organoid by day 60 was immunostained with Cntnap1 (green), CUX1 (red), and SOX2 (gray). CTIP2, a marker of cortical plate layer V; CUX1, a marker of layer II. Scale bar: 50 μm.

Since studies on the function of Cntnap1 have always focused on myelination, which is principally a postterm event, few studies have revealed the expression pattern of CNTNAP1 in early CNS development (35, 36). Wu et al. found that CNTNAP1 immunoreactivity is predominantly expressed in the mouse cerebral cortex during the perinatal period (E12–P0), especially in SOX2/Nestin^+^ RGCs and βIII-tubulin^+^ cortical neurons (12). To investigate the expression pattern of *Cntnap1* mRNA in the cortex during early development, we examined the RNA level of *Cntnap1* in the mouse cortex during the perinatal period via quantitative PCR (qPCR) (*n* = 3 mice for each group). We found that *Cntnap1* was markedly increased in the mouse cortex during E12–P7 (Figure 3A). Then, we collected total RNA of primary neurons cultured in vitro from day 0 to day 16 to measure the RNA expression level of *Cntnap1* (Figure 3B). Costaining for CNTNAP1 and the neuronal somatodendritic marker MAP2 showed increased expression of CNTNAP1 in the MAP2^+^ neurons in vitro (Figure 3D). Together with the IHC results from primary cortical neurons in vitro, these results revealed that *Cntnap1* expression was increased, especially after day 8 (Figure 3, B and D).

We would to further confirm that CNTNAP1 is expressed in early human brain. However, the actual human brain samples are hard to acquire. Therefore, we decide to use organoids derived from human induced pluripotent stem cells (iPSCs) to examine the CNTNAP1 expression. The experiment using human brain organoids is to further validate the expression of CNTNAP1 in early human brain development. We collected human iPSC–derived organoids to perform immunostaining experiments with CNTNAP1, CTIP2, CUX1, and SOX2 antibodies. The development of forebrain organoid recapitulates the inside-out pattern of the human cortical development (35, 37). CTIP2 (a marker of layer V) was identified around day 30, and CUX1 (a marker of layer II) was identified around day 60 (Figure 3E). Therefore, day 30 to day 60 is the main period during which the 6-layered cortical plate (CP) begins to form and develop. In order to further confirm the expression of CNTNAP1 in the human brain, we measured the RNA level of *CNTNAP1* of normal human iPSC–derived forebrain organoids from day 0 to day 60 and expression patterns. We found that *CNTNAP1* expression was also increased, especially after day 30 (Figure 3, C and E). These results suggest a role for *Cntnap1* in cortical radial migration.

### Cntnap1 KD interferes the radial migration and neurite morphogenesis of cortical neurons.

**Figure 4:**
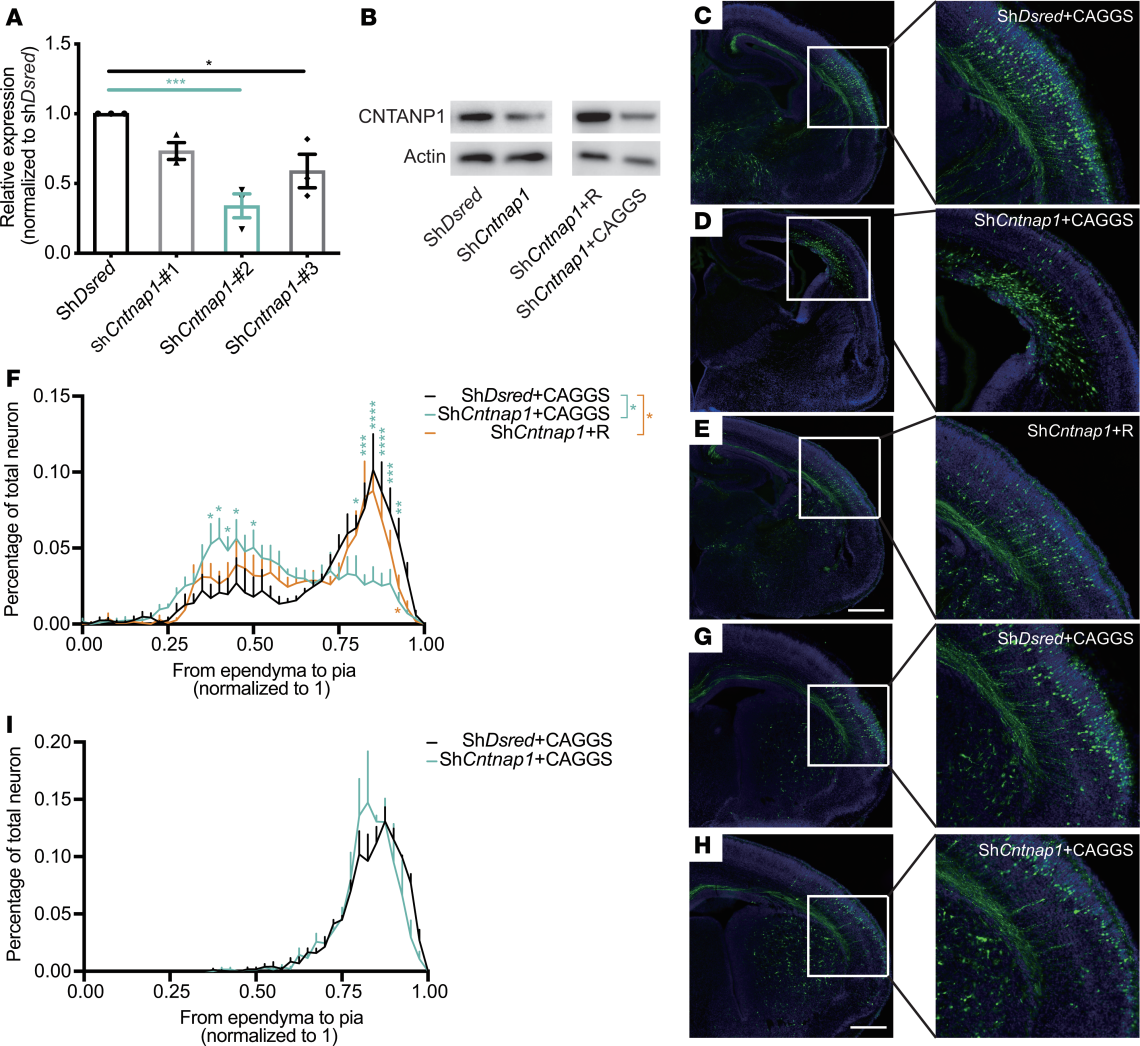
Knockdown of Cntnap1 delays neuronal migration in vivo. (**A**) The relative *Cntnap1* mRNA expression levels were analyzed by real-time RT-PCR, and Sh*Cntnap1*-#2 was selected as the most effective. Cultured mouse cortical neurons were electrotransfected with *Cntnap1* shRNA constructs #1, #2, or #3 at 0DIV and then subject to FACS at 7DIV. Statistical significance was evaluated by ordinary 1-way ANOVA, ***P = 0.001, *P = 0.0165. Data are shown as mean ± SEM. All data were collected from 3 independent experiments. (**B**) On the left is a representative Western blot of cultured mouse primary cortical neurons electrotransfected with shDsred versus sh*Cntnap1-*#2 after FACS at 7DIV; on the right is a representative Western blot of cultured mouse primary cortical neurons electrotransfected with sh*Cntnap1-*#2 + R versus sh*Cntnap1-*#2 + CAGGS after FACS at 7DIV. (**C**–**E**) Representative images of coronal slices of E18.5 mouse brains transfected with the indicated constructs by in utero electroporation at E13.5. At E18.5, cortical neurons transfected with sh*Cntnap1-*#2 + CAGGS were present in the VZ/SVZ instead of the CP. Transfected cells were visualized by staining coronal slices with a GFP antibody. Scale bar: 500 μm. (**F**) Quantitative analysis of the distribution of cortical neurons transfected with different constructs from the ependyma to the pia mater (5 mice from each condition were randomly selected and measured). Statistical significance was evaluated by 2-way ANOVA. Data are shown as mean ± SEM. *P* = 0.1234 (ns); ****P < 0.0001, ***P < 0.001, **P < 0.01, *P < 0.05. All data were collected from 3 independent experiments. (**G** and **H**) Representative images of coronal slices of P0 mouse brains transfected with the indicated constructs by in utero electroporation at E13.5. By P0, cortical neurons had migrated normally from the SVZ into the cortex in both control- and Sh*Cntnap1-*#2–transfected brains. Scale bar: 500 μm. (**I**) Quantitative analysis of the distribution of cortical neurons transfected with different constructs from the ependyma to the pia mater (*n* = 3 for each group). Statistical significance was evaluated by 1-way ANOVA. Data are shown as mean ± SEM. All data were collected from 3 independent experiments.

The migration of newly born neurons is a precisely regulated process that is critical for the development of brain architecture. To investigate the role of *Cntnap1* in cortical neuron migration, we knocked down *Cntnap1* expression in mouse pyramidal neural progenitors using in utero electroporation. We designed 4 fugw-H1-shRNA-GFP constructs, including sh*Cntnap1*-#1, -#2 and -#3, which targeted *Cntnap1*, and a control construct (shDsred). The fugw-H1-shRNA-GFP construct encoded GFP in a separate reading frame, allowing the tracing of electroporated cells in vitro and in vivo. We electrotransfected cortical neurons isolated from E14.5 C57BL/6J mice with these 4 constructs. After FACS at 7 days in vitro (7DIV), we tested the KD efficiency at the RNA level by real-time reverse transcription PCR (real-time RT-PCR). Compared with the control shRNA, the sh*Cntnap1*-#2 resulted in a significant decrease in the RNA level of *Cntnap1* in GFP^+^ neurons (Figure 4A). We also cloned the ORF of the mouse WT *Cntnap1* gene into the CAGGS-Flag-GFP vector (WT), and then we performed synonymous point mutagenesis to create a rescue construct (*Cntnap1*-R) against sh*Cntnap1*-#2. To validate the results at the protein level, we transfected shDsred versus sh*Cntnap1*-#2 and *Cntnap1*-R plus sh*Cntnap1* versus sh*Cntnap1*-#2 plus CAGGS (an empty CAGGS vector) into neurons and then collected cells after FACS at 7DIV. We found that the in vitro genetic manipulation of sh*Cntnap1*-#2 and *Cntnap1*-R was efficient at the protein level (Figure 4B).

Through the in utero electroporation of constructs (shDsred plus CAGGS, sh*Cntnap1* plus CAGGS, or sh*Cntnap1* plus *Cntnap1*-R) into the E13.5 mouse brain, we genetically manipulated pyramidal neural progenitors and migrating neurons. The distribution of GFP-labeled cells in the neocortex was examined at E18.5. While the majority of GFP-labeled cells in the control group left the ventricular zone/subventricular zone (VZ/SVZ) and some of them had already arrived at the terminals, a large percentage of GFP^+^ cells of the shRNA group remained in the VZ/SVZ (Figure 4, C, D, and F). The cotransfection of *Cntnap1*-R partially rescued this change (Figure 4, E and F). However, the ratio of GFP^+^ cells that reached the upper CP was comparable between the sh*Cntnap1*-#2 and control groups at P0 (Figure 4, G–I). These results indicate that *Cntnap1* deficiency delays neuronal migration during perinatal cortical development.

The binding of contactin to CNTNAP1 in cis interactions in the plasma membrane is required for developmental events such as neurite growth, neural cell adhesion, myelination, and neuronal migration (19, 38, 39). In *Cntn1*-KO mice, axonal guidance and dendritic projections in granule cells are defective (40). We therefore knocked down *Cntnap1* expression in primary neurons to determine whether CNTNAP1 is involved in neurite genesis and arborization.

**Figure 5:**
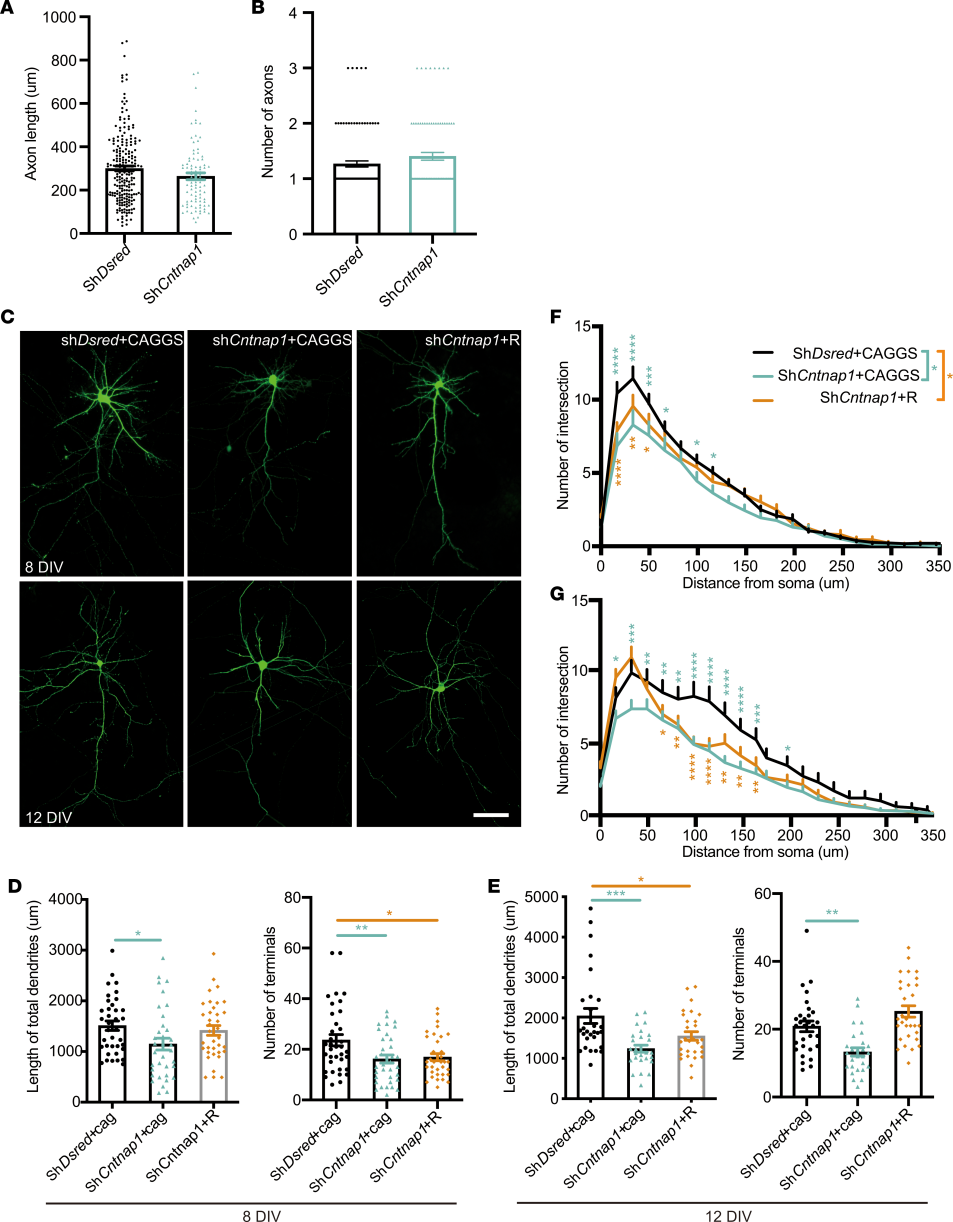
*Cntnap1* regulates dendritic arborization in vitro. (**A** and **B**) Quantification of the total axon length and the number of terminals of neurons transfected with the indicated constructs (214 and 89 neurons from each group were randomly selected and measured.). All data were collected from 3 independent experiments. Statistical significance was evaluated by Student’s *t* test. Data are shown as mean ± SEM. For the quantification of total axon length, *P* = 0.0736; for number of terminals, *P* = 0.1163. (**C**) Representative images of 8DIV and 12DIV cultured neurons transfected with the indicated plasmids. The constructs were transfected at 1DIV using Lipofectamine 2000 and immunolabeled with anti-GFP at 8DIV and 12DIV. Scale bar: 50 μm. (**D** and **E**) Quantification of the total dendrite length and the number of terminals of 8DIV and 12DIV neurons transfected with the indicated constructs. For 8DIV, 37, 36, and 36 neurons from each group were randomly selected and measured. Statistical significance was evaluated by 1-way ANOVA. For total dendritic length, *P = 0.0354; for the quantification of dendritic terminals, **P = 0.0069 and *P = 0.0171. For 12DIV, 29, 27, and 29 neurons from each group were randomly selected and measured. Statistical significance was evaluated by 1-way ANOVA. ***P = 0.0001, **P = 0.0016, and *P = 0.02. All data were collected from 3 independent experiments. (**F** and **G**) Quantification of the dendritic branching of neurons collected at 8DIV (**F**) and 12DIV (**G**), as determined by Sholl analysis (30 and 38 neurons from each group were randomly selected and measured). *P* = 0.1234 (ns); ****P < 0.0001, ***P < 0.001, **P < 0.01, *P < 0.05. Statistical significance was evaluated by 2-way ANOVA, Data are shown as mean ± SEM. All data mentioned above were collected from 3 independent experiments

To assess the function of *Cntnap1* in neurite genesis, we transfected shDsred and sh*Cntnap1*-#2 into neurons at 1DIV and then performed anti-GFP immunostaining at 3DIV. There was no significant difference in total axon length or the number of terminals between the control- and sh*Cntnap1*-#2–transfected cells (Figure 5, A and B). To assess the function of *Cntnap1* in dendritic outgrowth and branching in vitro, we examined the effects of *Cntnap1* KD by cotransfecting primary neurons with shDsred plus CAGGS, sh*Cntnap1*-#2 plus CAGGS, or sh*Cntnap1*-#2 plus *Cntnap1*-R. Neurons were collected at 8DIV and 12DIV. The total dendrite length and the number of terminals were significantly reduced in neurons expressing the sh*Cntnap1*-#2 construct compared with the control at 8DIV and 12DIV (Figure 5, C–E). Then, using Sholl analysis (Fiji, ImageJ, NIH), we found that the intersections of neurons transfected with sh*Cntnap1*-#2 were also affected compared with those of the control (Figure 5, F and G). It is worth noting that dendritic morphology, including the total length, total number of terminals, and intersections, of neurons collected at 12DIV was more affected than that of neurons collected at 8DIV. This is consistent with the pattern of increasing expression of *Cntnap1* during in vitro neuronal development. The effects of sh*Cntnap1*-#2 were rescued by the cotransfection of *Cntnap1*-R. These results indicate that *Cntnap1* is required for neurite genesis and development, which are critical for neuronal migration and, thus, the formation of functional neural circuitry in the brain.

### Cntnap1^–/–^ mice showed delay migration and sparser dendrite branches.

**Figure 6:**
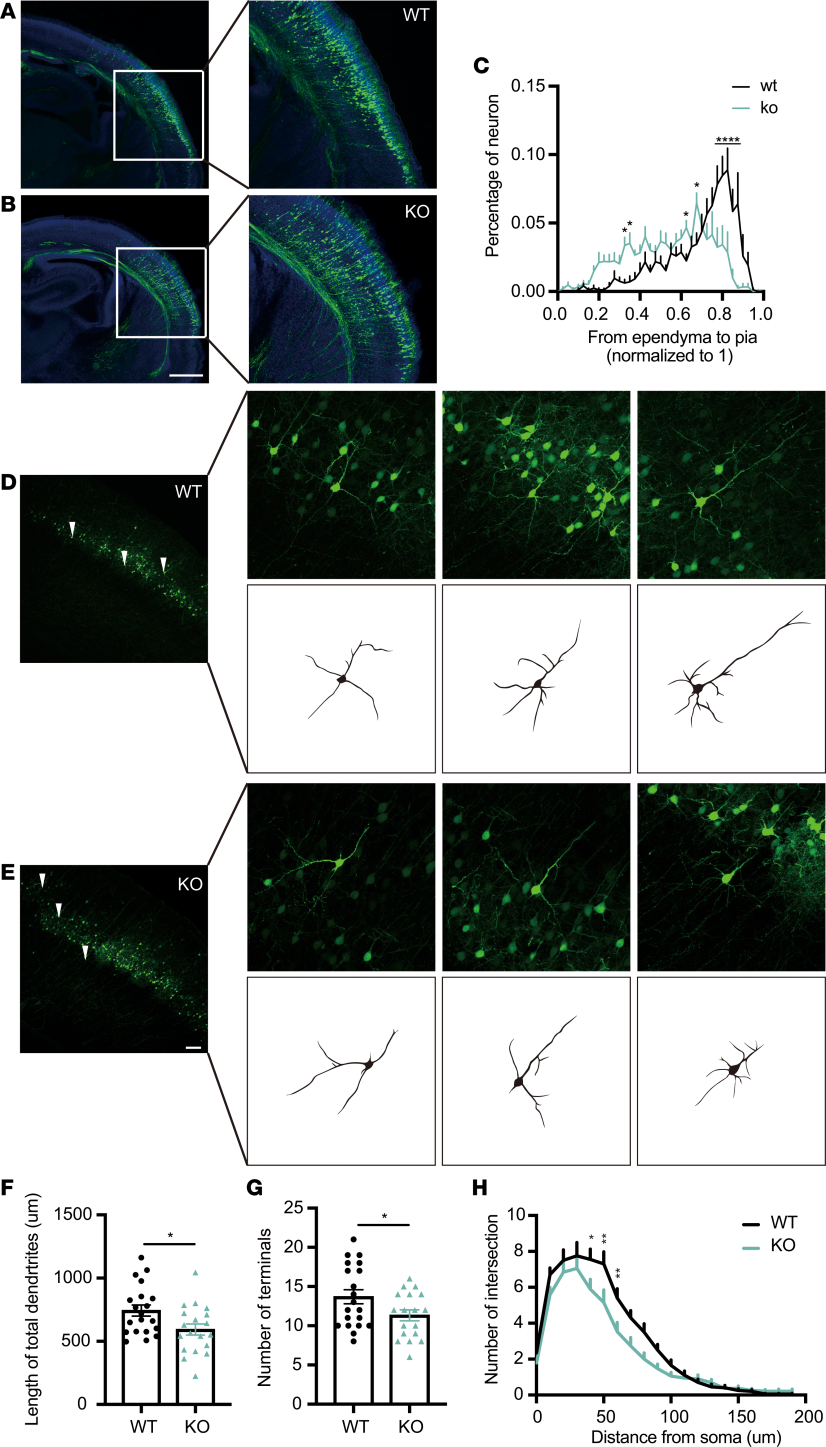
*Cntnap1^–/–^* mice showed delay migration and sparser dendrite branches. (**A** and **B**) Representative images of coronal slices of E18.5 mouse brains transfected with shDsred at E13.5. At E18.5, KO cortical neurons showed a delay of migration compared with WT. Transfected cells were visualized by staining coronal slices with a GFP antibody. Scale bar: 500 μm. (**C**) Quantitative analysis of the distribution of cortical neurons from the ependyma to the pia mater (3 mice from each condition were randomly selected and measured). Statistical significance was evaluated by 1-way ANOVA. Data are shown as mean ± SEM. *P* = 0.1234 (ns). (**D** and **E**) Representative images of GFP-labeled cortical neurons in WT and KO mice by P10. We halved the plasmid concentration for sparse labeling. Scale bar: 100 μm. The neurite tracings of representative pyramidal neurons are shown on the right. (**F** and **G**) Quantification of the total dendrite length and the number of terminals of GFP labeled neurons. A total 20 and 19 neurons from WT and KO were randomly selected and measured. Statistical significance was evaluated by Student’s *t* test. Data are shown as mean ± SEM. *P* = 0.027 (**E**), *P* = 0.0462 (**F**). (**H**) Quantification of the dendritic branching of neurons collected at P10, as determined by Sholl analysis (20 and 19 neurons from each group were randomly selected and measured). *P* = 0.1234 (ns); ****P < 0.0001, ***P < 0.001, **P < 0.01, *P < 0.05. Statistical significance was evaluated by 2-way ANOVA. Data are shown as mean ± SEM.

In order to confirm the cortical development defect in Cntnap1-KO mice, we then constructed *Cntnap1*^–/–^ mice using CRISPR/Cas9 system targeting exon 13 of *Cntnap1*. Compared with WT littermates, the *Cntnap1*^–/–^ mice, similar to those constructed by Harris BS, et al. (41), presented decreased body size, hypotonia, wide-based gait, tremors, and generalized motor paresis. Although Cntnap1-KO mice are unhealthy, we still performed rigorous studies. We performed the CRISPR/Cas9-mediated gene deletion in fertilized eggs, followed by electroporation in embryonic-KO mice at E13.5 and harvested embryos at E18.5 to analyze the migration of cortical neuron. Similarly, we found a delayed migration of neuron in KO mice at E18.5 (Figure 6, A–C). However, whether in the cerebral cortex of KO mice or WT mice, all labeled neurons completed migration at P10 (Figure 6, D and E). Furthermore, the cortical neurons in KO mice exhibited shorter dendrites, fewer dendritic terminals, and sparser branches compared with WT (Figure 6, D–H). These results indicate that *Cntnap1* deficiency interferes with neuronal migration and neurite morphogenesis during perinatal cortical development.

### Cntnap1^–/–^ mice showed increased spine density and an increased number of PV^+^ neurons in the cortex and hippocampus.

**Figure 7:**
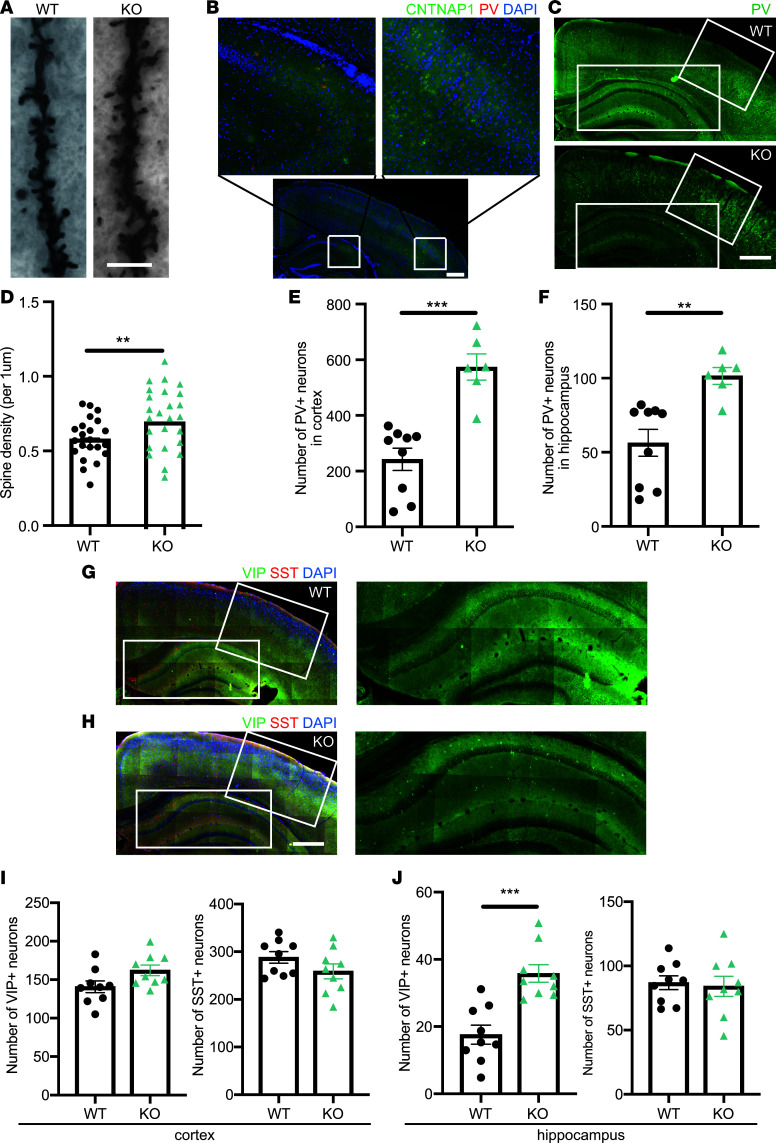
*Cntnap1*^–/–^ mice showed increased spine density and an increase in the number of PV^+^ and VIP^+^ neurons. (**A**) Representative Golgi staining of cortical layers II/III of adult Cntnap1^−/−^ mice (apical dendrites, 5 months of age, *n* = 3 for WT and Cntnap1^−/−^ mice). Scale bar: 4 μm. (**B**) Costaining of Cntnap1 and PV in WT mouse brain at P15. Scale bar: 200 μm. (**C**) Representative immunochemical images of the S1 primary somatosensory cortex and hippocampus in coronal slices of P15 mouse brains stained with a PV antibody. Scale bar: 500 μm. (**D**) Quantitative analysis of spine density in *Cntnap1*^–/–^ mice (22 and 25 neurons from each group were randomly selected and measured). Statistical significance was evaluated by Student’s *t* test. **P = 0.0093. Data are shown as mean ± SEM. (**E** and **F**) Quantitative analysis of the number of PV^+^ neurons in *Cntnap1*^–/–^ mice (*n* = 3 mice for WT and KO) compared with their normal littermates. Statistical significance was evaluated by Student’s *t* test. Data are shown as mean ± SEM. For the quantification of PV^+^ neurons in cortex, ***P = 0.001. For the quantification of PV^+^ neurons in hippocampus, **P = 0.0025. (**G** and **H**) Representative immunochemical images of the S1 primary somatosensory cortex and hippocampus in coronal slices of P15 mouse brains stained with a VIP (green) and SST (red) antibody. Scale bar: 500 μm. On the right are enlarged images of boxed hippocampus stained with VIP antibody. (**I** and **J**) Quantitative analysis of the number of VIP^+^ and SST^+^ neurons in *Cntnap1*^–/–^ mice compared with WT (*n* = 3 mice for WT and KO). For the quantification of VIP^+^ neurons in hippocampus, ***P = 0.0002. Statistical significance was evaluated by Student’s *t* test. Data are shown as mean ± SEM.

Dendritic spines are a major site of synaptic contacts. Through Golgi staining, we found that the spine density of cortical neurons was increased in *Cntnap1*^–/–^ mice in comparison with WT mice, indicating that *Cntnap1* deficiency leads to an abnormal synapse development in vivo (Figure 7, A and D). Aberrant spine density might contribute to increased excitability and a tendency to develop seizures. Among the 11 patients with available EEG data mentioned above, 63% (5 of 8) who survived beyond infancy (>1 year) had seizures. One patient developed generalized clonic, brief tonic, and myoclonic seizures at the age of 10 weeks, suggesting the need for early EEG tests in suspected *CNTNAP1*-related disease patients.

Interestingly, *Cntnap1*^–/–^ mice showed a marked increase in the number of PV^+^ GABAergic neurons in the cortex and hippocampus compared with that in WT littermates at P15 (Figure 7, B, C, E, and F). Additionally, VIP^+^ GABAergic neurons were increased in the hippocampus of *Cntnap1*^–/–^ mice compared with 3 WT P15 mice (Figure 7, G–J). These data suggest that *CNTNAP1* may play a role in the development of GABAergic neurons during the early postnatal period. Interestingly, Tong et al. also found that *Cntnap*3^–/–^ mice showed increased PV^+^ neurons in the cortex and hippocampus (7), while Peñagarikano et al. found a decreased interneuronal density in *Cntnap*2^–/–^ mice (9). These results suggest a role for the CNTNAP family in balancing neocortical excitation/inhibition during early development.

## Discussion

Several studies have demonstrated the critical role of CNTNAP1 in axoglial interactions in the nervous system since it was first identified in 1997 (14). *Cntnap1*-KO mice exhibit tremors, paresis, ataxia, and failed formation of normal paranodal junctions (2). Therefore, *CNTNAP1* deficiency is thought to cause hypomyelinating neuropathy. Myelination of the CNS occurs relatively late in brain development in humans and peaks around 2 years of age (42). Typical hypomyelinating diseases such as PMDs and Alexander disease feature an uneventful pregnancy and a short period of normal development before a subsequent loss of milestones, which is consistent with the timing of myelination.

With the rapid development of NGS, patients with pathogenic *CNTNAP1* mutations have been reported recently. All of these patients present hypomyelination and hypotonia, which are consistent with previous animal findings. However, the prenatal and massive neonatal symptomatology, including brain atrophy and lethal overall disease course, suggests a strong neuronal pathology.

We found that CNTNAP1 deficiency significantly affected dendritic branching and elongation in vitro and in vivo, while axon genesis was not affected (Figure 3, B and D, and Figure 5, C–G). However, Devanathan et al. (17) compared neurite outgrowth in the cerebellar neurons of *Cntnap1*^–/–^ and *Cntnap1*^+/+^ mice maintained on glass coverslips. After 24 hours of plating, the neurites of *Cntnap1*^–/–^ neurons were longer than those of *Cntnap1*^+/+^ neurons. This suggests that CNTNAP1 inhibits neurite outgrowth in cerebellar neurons in vitro. One possible explanation for this inconsistency is that CNTNAP1 may play a different role in the process of developing neurite outgrowth and adult cerebellar neurite regeneration after injury. Taken together, CNTNAP1 might have a critical role in neurite outgrowth and arborization. Since alterations in dendrite genesis and branching patterns contribute to several neurodevelopmental disorders, such as ASDs, Down syndrome (DS), Fragile X syndrome (FXS), and Rett syndrome (RS) (43). CNTNAP1 deficiency might contribute to the global cognitive deficits found in *CNTNAP1*-related disease patients.

Through the in utero electroporation of sh*Cntnap1*-#2 into the embryonic brain at E13.5, we found that *Cntnap1* KD led to a transient delay in neuronal radial migration (Figure 4). In addition, we confirmed this transient migration defects in *Cntnap1*-KO mice (Figure 6, A and B). Although the migration of *Cntnap1*-deficient neurons was found to reach the normal level after birth, a transient migration delay during essential developmental stages is possibly associated with the perturbed establishment of neural circuits. Wu et al. (12) also found a transient reduction in neurons in layers II–IV of the *Cntnap1*^–/–^ CP at E17.5, and that the number of neurons reached the control level at P0. However, they suggested that deficiency of CNTNAP1 in neural progenitor cells (NPCs) results in delayed production of cortical neurons through Notch signaling. Taken together, *Cntnap1* deficiency results in aberrant cortical neuron development.

In conclusion, *Cntnap1* is highly enriched in the developing cortical neurons in humans and mice. It plays a critical role in regulating neuronal migration, neurite growth, and spine density, as well as the development of GABAergic neurons. Our findings suggest that *Cntnap1* deficiency in humans may also result in defects of neuronal morphogenesis and migration during a crucial stage of early brain development and that this impaired process may contribute to the pathophysiology of disease phenotypes.

## Methods

### Human sample collection

Genomic DNA was extracted from the peripheral blood of the participants with a QIAamp DNA Blood Mini Kit (QIAGEN) following the manufacturer’s specifications. DNA fragments were enriched, and WES was performed using the Agilent SureSelectXT Human All Exon 50 Mb kit.

### WES and Sanger sequencing validation

Genomic DNA fragments of the 2 patients were enriched for WES through the Agilent SureSelectXT Human All Exon 50Mb kit. DNA fragments were ligated with adaptors, and 2 paired-end DNA libraries with an insert size of 500 bp were formed. A HiSeq 2500 sequencer was used following the manufacturer’s instructions (Illumina). We aligned clean reads to the reference human genome (GRCh37/hg19) using the Burrows-Wheeler Aligner (BWA) (v.0.5.9- r16). Subsequently, SAMtools and Picard were used for sorting, merging, and removing duplication for the BAM files (http://broadinstitute.github.io/picard/). Variants were called using GATK. The mean coverage sequencing depth on the official target ranged from 109.83 to 161.68. The mapping rate of data ranged from 99.90% to 99.92%, and the average coverage of the target region was from 99.80% to 99.91%. The candidate variants were validated using PCR, and the PCR-amplified DNA products were subjected to direct automated sequencing (3500XL Genetic Analyzer, Applied Biosystems) according to the manufacturer’s instructions.

### Plasmids

The human-*CNTNAP1* (NM_003632.3) CDS was amplified from human iPSC–derived neurons, and the mouse-*Cntnap1* (NM_016782.2) CDS was amplified from mouse primary neurons by KOD FX (www.toyobo.co.jp/e/bio). The human-*CNTNAP1* CDS and mouse-*Cntnap1* CDS (coding sequences) were inserted into the pCAGGS vector. Both constructs were validated by sequencing. The primers were as follows. Human *CNTNAP1* CDS PCR, forward: 5′-GGCGGCATCGATATGATGCATCTCCGGCTCTT-3′; human *CNTNAP1* CDS PCR, reverse: 5′-GGCGGCGAGCTCTTCAGACCTGGACTCCTCC-3′.

The Flag-tag were synthesized as oligonucleotide primers and manually annealed; they were then inserted into human *CNTNAP1*–CAGGS vector resulting to a fusion CNTNAP1 protein with Flag. The primers were as follows. Flag insert oligo, forward: 5′-CGATTACAAGGATGACGACGATAAGTGAGAGCT-3′; Flag insert oligo, reverse: 5′-CTCACTTATCGTCGTCATCCTTGTAATCGAGCT-3′.

The sh*CNTNAP1* (#1, #2, #3, and Dsred) siRNAs were synthesized as oligonucleotide primers and manually annealed. Primers were as follows. sh*CNTNAP1*-#1 oligo, forward: 5′-CTAGAGCATCTATGGTTGTCCCTATTCAAGAGATAGGGACAACCATAGATGCTTTTTG-3′; sh*CNTNAP1*-#1 oligo, reverse: 5′-GATCCAAAAAGCATCTATGGTTGTCCCTATCTCTTGAATAGGGACAACCATAGATGCT-3′; sh*CNTNAP1*-#2 oligo, forward: 5′-CTAGACCCATCACTTTGTGCTCAATTCAAGAGATTGAGCACAAAGTGATGGGTTTTTG-3′; sh*CNTNAP1*-#2 oligo, reverse: 5′- GATCCAAAAACCCATCACTTTGTGCTCAATCTCTTGAATTGAGCACAAAGTGATGGGT-3′; sh*CNTNAP1*-#2 oligo, forward: 5′- CTAGAGCACAGGGACTTCATACTTTTCAAGAGAAAGTATGAAGTCCCTGTGCTTTTTG-3′; sh*CNTNAP1*-#2 oligo, reverse: 5′- GATCCAAAAAGCACAGGGACTTCATACTTTCTCTTGAAAAGTATGAAGTCCCTGTGCT-3′; Dsred siRNA oligo, forward: 5′- CTAGAAGTTCCAGTACGGCTCCAATTCAAGAGATTGGAGCCGTACTGGAACTTTTTTG-3′; Dsred siRNA oligo, reverse: 5′- GATCCAAAAAAGTTCCAGTACGGCTCCAATCTCTTGAATTGGAGCCGTACTGGAACTT-3′.

The sh*CNTNAP1* (#1, #2, #3, and Dsred) siRNA sequences were inserted into the FUGW vector (Addgene, 14883).

### RNA isolation and reverse transcription

C57BL/6J mice were sacrificed after being deeply anesthetized with 0.14 g/kg sodium pentobarbital. The brains were quickly placed in precooled 4°C PBS (Corning, 21–040-CVR). The cortical region of the brain was isolated in PBS solution under the guidance of The Mouse Brain in Stereotaxic Coordinates (44). A total of 0.1 g of each cerebral cortex was placed in 1 mL Trizol (Invitrogen, 15596018) and then homogenized thoroughly in an ice water bath. Total RNA was extracted following the manufacturer’s instructions (TRIzol reagent). The RNA was reverse transcribed using the Reverse Transcriptase M-MLV kit (Takara, D2639B). Total mRNA (1 μg) and 50 nmol oligo-dT were used for reverse transcription.

### qPCR

qPCR was performed to investigate the mRNA level of *Cntnap1* using SYBR green (Toyobo, QPK-201). Mouse Rpl191 and Human GAPDH were used for normalization as an internal control, respectively. The following primers were used. Mouse *Cntnap1* RT, forward: 5′-ACTTCCCGCTGACAGAACAGAAGTT-3′; mouse *Cntnap1* RT, reverse: 5′-CGAGGGGTTCGGAAATGGGTCTT-3′; mouse Rpl191 RT, forward: 5′-AAGCCTGTGACTGTCCATTC-3′; mouse Rpl191 RT, reverse: 5′-CTTCTTGGATTCCCGGTATC-3′; human *CNTNAP1* RT, forward: 5′-TCAAGGTGGATGGTCAACTGG-3′; human *CNTNAP1* RT, reverse: 5′-AGCCGATAAGCCTCACAGGA-3′; human GAPDH RT, forward: 5′-TGATGACATCAAGAAGGTGGTGAAG-3′; human GAPDH RT, reverse: 5′-TCCTTGGAGGCCATGTGGGCCAT-3′.

The qPCR program was set as follows: 95°C denaturation for 10 minutes, followed by 40 cycles of 95°C for 10 seconds, 60°C for 15 seconds, and 72°C for 20 seconds. The *CNTNAP1* expression level was calculated and standardized using the ΔCt method and the internal control mentioned above.

### Western blot analysis

Protein samples were harvested from cultured cortical neurons, run on an 8% SDS-polyacrylamide gel at a constant voltage (80 V for 30 minutes and 120 V for 90 minutes), and then transferred onto PVDF membranes for 90 minutes at 250 mA (MilliporeSigma; pore size, 0.45 μm). The blots were blocked in TBST solution with 5% BSA for 2 hours at room temperature (RT) and then incubated with primary antibody overnight at 4°C. After washing with TBST, the blots were treated with secondary antibody for 2 hours at RT. The protein bands were detected by chemiluminescence (ECL Western Blotting Substrate, Pierce, 32106). The antibodies that used were as follows: anti–β-actin (rabbit), 1:1000 (Cell Signaling Technology [CST], 4967); anti-Flag (mouse), 1:1000 (Abmart, M20008); anti-CNTNAP1 (rabbit), 1:1000 (Abcam, 133634).

### Primary culture of cortical neurons and transfection

Mouse cortical neurons were cultured from E13.5 mouse embryos (45). The cerebral cortices were dissected and digested with 20 U/mL papain (Worthington, LS003126) at 37°C for 30 minutes. Neurons were dissociated into suspension and then electrotransfected with constructs in Nucleofector solution (Lonza) buffer with an AMAXA Nucleofector (AAD-1001S) machine before plating. The AMAXA Mouse Neuron Nucleofector kit was used under the “mouse, neuron, 0–005” program, and the neuronal viability was over 80%. Neurons were plated and cultured on Lab-Tek II chamber slides (Thermo Fisher Scientific, 154941) at a density of 80,000 cells/well in neurobasal medium (Thermo Fisher Scientific, 21103-049) with 0.2% B27 (Thermo Fisher Scientific, 17504-044) and 2 mM Glutamax-I (Thermo Fisher Scientific, 35050-061). Transfected neurons were mixed with untreated neurons at a ratio 1:8 for sparse labeling. Half of the medium was changed every 2 days. The neurons were fixed at 4DIV (for axon experiments) or 8DIV or 12DIV (for dendrite experiments) for immunofluorescence staining; fixed at 0DIV, 4DIV, 8DIV, 12DIV for immunoblotting (for expression pattern experiments); or harvested at 0DIV, 4DIV, 8DIV, 12DIV, or 16DIV for qPCR after electrotransfection.

### In utero electroporation

In utero electroporation was performed as described with a few modifications (46). Briefly, pregnant C57BL/6J (RRID: IMSR_JAX:000664; The Jackson Laboratory) mice were subjected to abdominal incision at E13.5 to expose the uterus after being anesthetized by i.p. injection of 0.7% sodium pentobarbital at a dose of 10 μL/g. Plasmids (2 μg/μL) with 0.05% Fast Green (MilliporeSigma) were injected into the lateral ventricles of embryonic brains using a glass micropipette. Electrical pulses were then delivered to the embryos by the electrodes of an Electro Square Porator (ECM 830, BTX). Pulses were applied for 50 ms at 30 V at 1-second intervals. Postsurgery animals were placed on a thermostatic electric blanket (26°C) until they recovered from anesthesia. The mice were sacrificed at E18.5 or P0, and then the pups’ brains were quickly removed and fixed in 4% PFA for 24 hours. The fixed brains were dehydrated in 15% and 30% sucrose solution for 1 day, separately. The brains were embedded in OCT, and longitudinal brain sections (40 μm) were obtained with a Leica freezing microtome (CM1950).

### Animals

All mice were the experimental model in this study and were maintained in a specific pathogen–free (SPF) facility under automatically controlled temperature, humidity, ventilation, and light conditions (light, 0:00 to 12:00; dark, 12:00 to 24:00). *Cntnap1*-KO mice were generated as previously described (47). Harris BS, et al. constructed a *Cntnap1^shm–5j^* mouse allele (41) through a spontaneous point mutation (c. T2029C, p.S674P) within exon 13. Therefore, 4 sgRNAs spaced 10–200 bp targeting sites at the exon 13 of the *Cntnap1* gene were designed and coinjected with *Cas9* mRNA into zygotes, resulting in more frequent complete gene KO on the B6D2F1 genetic background. The sequences of the small guide RNAs (sgRNAs) used for KO were as follows. *Cntnap1* gRNA1: 5′-accgagcgtggacagttgtg-3′; *Cntnap1* gRNA2: 5′-ggaggcattccagtattgga-3′; *Cntnap1* gRNA3: 5′-cttcccaacactgtgagcag-3′; *Cntnap1* gRNA4: 5′-acctgcagtgttgagcagcc-3′.

The genotypes of all mice used in the experiments were confirmed by Sanger sequencing. The regions including the sgRNAs directing sites were amplified by primers as follows: *Cntnap1* Genotyping first, forward: 5′-ACTGCGAACTTACCGGCTAC-3′; *Cntnap1* Genotyping first, reverse: 5′-GTTCCTCATTGCGGCCAATC-3′; *Cntnap1* Genotyping second, forward: 5′-GGGAAGGTTGTTGGGGATCA-3′; *Cntnap1* Genotyping second, reverse: 5′-ATTGCGGCCAATCCAAAAGC-3′.

The mice used for PV staining were P15 mice. The mice used for Golgi staining were 5 months old.

### Generation of cerebral organoids from human iPSCs

Cerebral organoids were generated as previously described (48). Briefly, human iPSCs were lifted from the plates using Dispase II (Invitrogen) and cultured with Neural Induction Medium (NIM) consisting of DMEM/F12, 1× N2 supplements (Invitrogen), plus DMH1 (2 μM, Tocris) and SB-431542 (2 μM, Stemgent). For the first 4 days, NIM was changed every day and supplemented with DMH1 and SB-431542. On days 5 and 6, NIM was changed each day and supplemented with SB-431542 and CHIR99021 (1 μM, Tocris). On day 7, organoids were embedded in Matrigel (BD Biosciences) and cultured with NIM, plus SB-431542 and CHIR99021, for 6 days. On day 14, embedded organoids were dissociated from Matrigel and cultured with differentiation medium, consisting of DMEM/F12, N2, and B27 supplements (Invitrogen), 0.1 mM β-mercaptoethanol (MilliporeSigma), 1× nonessential amino acids, and 2.5 μg/mL insulin (MilliporeSigma).

### Immunohistochemical staining

#### Cultured neurons.

After washing with PBS for 5 minutes, neurons were fixed in 4% PFA for 20 minutes at RT. The neurons were washed 3 times with PBS and then blocked (3% BSA and 0.1% Triton X-100 in PBS) for 1 hour at RT. The neurons were incubated with primary antibody in blocking buffer overnight at 4°C. After washing 3 times with PBS, the neurons were incubated with secondary antibody and 1:500 DAPI in antibody buffer for 2 hours at RT. Finally, the neurons were washed 3 times with PBS.

#### Slices.

After washing with PBS for 5 minutes, sections were blocked (5% BSA and 0.3% Triton X-100 in PBS) for 2 hours at RT. After incubation with primary antibody in antibody buffer (3% BSA and 0.1% Triton X-100 in PBS) overnight at 4°C, the sections were washed 3 times with PBS and then incubated with secondary antibodies and 1:500 DAPI for 2 hours at RT. The sections were then washed 3 times with PBS.

The antibodies that were used were as follows: anti-GFP (rabbit), 1:1000 (Invitrogen, A11122); anti-PV (mouse), 1:1000 (MilliporeSigma, MAB157); anti-MAP2 (mouse), 1:2000 (MilliporeSigma, MAB3418); anti-CNTNAP1 (rabbit), 1:200 (Abcam, 133634); anti-SOX2 (goat), 1:500 (R&D, AF2018); anti-CTIP2 (rat), 1:300 (Abcam, ab18465); anti-CUX1 (mouse), 1:500 (Santa Cruz Biotechnology Inc., sc-514008); anti-SST (mouse), 1:400 (Santa Cruz Biotechnology Inc., sc-55565); and anti-VIP (rabbit), 1:400 (CST, 63269).

For the axon and dendrite experiments, the total axonal length, dendritic lengths, number of dendritic terminals, and dendritic intersections were analyzed by ImageJ (RRID:SCR_003070; NIH). The chamber slides were randomly labeled and recorded by person A. Therefore, sample identifications were blinded to person B during data analysis. Length from the soma to the neurite terminal were determined as axonal and dendrite length.

For neuronal radial migration experiments, plasmids were randomly labeled and recorded by person A before in utero electroporation. Therefore, sample identifications were blinded to person B during following process.

### Golgi staining

The mice were deeply anesthetized (0.14 g/kg sodium pentobarbital) before sacrifice. Mouse brains were removed and rinsed quickly in double-distilled water to remove blood from the surface. Then, the brains were immersed in Golgi staining buffer (1:1 solution A/B) 1 day before according to the guidelines of the FD Rapid GolgiStainTM Kit (FD NeuroTechnologies, PK401). The Golgi staining buffer was changed in the first 24 hours, and then staining was performed for another 13 days. Then, the brains were immersed in solution C for another 5 days. The sections were cut at a thickness of 100 μm, and the slices were collected on gelatin-coated glass slides. We used 3 mice per group, and at least 18 slices from each mouse brain were obtained. The dendrite involved in spine density analysis was required to be > 20 μm.

### Statistics

Statistical tests were performed using GraphPad Prism 8 (version 8.1.1 for Mac, GraphPad Software, www.graphpad.com). Two-tailed Student’s *t* tests were used for analyses between 2 groups, 1-way ANOVA followed by Tukey’s multiple comparison tests were used for analyses between 3 groups, and 2-way ANOVA followed by Dunnett’s multiple comparisons tests were used for the curves (Sholl analysis and migration pattern). All data are shown as the mean ± SEM. *P* = 0.1234 (ns); ****P < 0.0001, ***P < 0.001, **P < 0.01, *P < 0.05. We used at least 3 mice from 2 different litters for the neuronal morphological experiments, and the in vitro experiments were performed independently at least 3 times.

### Study approval

This study was approved by the ethics committee of Children’s Hospital, Fudan University (2015-130 and 2016-235). All samples in this study were collected with appropriate informed consent. All animal experiments were performed with the approval of the Biomedical Research Ethics Committee at the Shanghai Institutes for Biological Science and following the guidelines of this committee.

## Author contributions

Study concept and design were contributed by ZQ and WZ. Experiment and data analysis were contributed by WL, CT and KL. Acquisition of clinical information was contributed by WL, WZ, LY, HW, and KY. Interpretation of WES data was contributed by WZ and YL. Drafting of the manuscript was contributed by WL. Critical revision of the manuscript was contributed by WZ and ZQ. ZQ and WZ obtained funding. Study supervision was contributed by ZQ and WZ.

## Figures and Tables

**Table 1:**
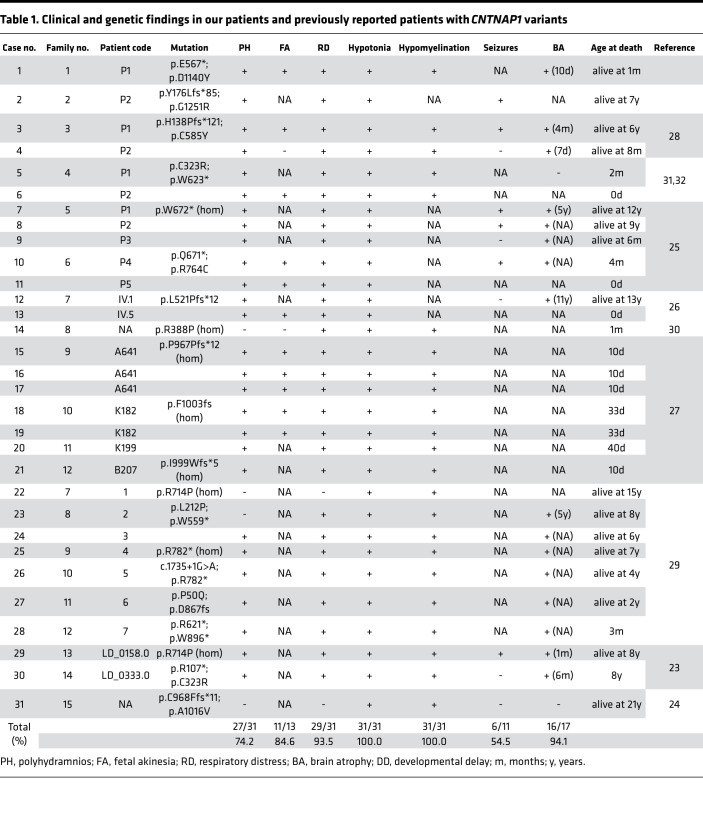
Clinical and genetic findings in our patients and previously reported patients with *CNTNAP1* variants

## Version 1 11/05/2020

Electronic publication
